# Microencapsulated sodium butyrate administered to patients with diverticulosis decreases incidence of diverticulitis—a prospective randomized study

**DOI:** 10.1007/s00384-013-1807-5

**Published:** 2013-12-18

**Authors:** Lukasz Krokowicz, Zoran Stojcev, Bartosz Filip Kaczmarek, Wojciech Kociemba, Elżbieta Kaczmarek, Jaroslaw Walkowiak, Piotr Krokowicz, Michal Drews, Tomasz Banasiewicz

**Affiliations:** 1 Department of General Surgery, Oncologic Gastroenterological and Plastic Surgery, Poznań University of Medical Sciences, ul. Przybyszewskiego 49, 60-355 Poznań, Poland; 2 Department of General, Vascular and Oncologic Surgery, Regional Hospital, Słupsk, Poland; 3 Department of Oncologic Surgery, Medical University, Gdańsk, Poland; 4 Department of Urology, Holy Family Hospital, SPZOZ Nad Matką i Dzieckiem, Poznań, Poland; 5 Department of Neuroradiology, Poznań University of Medical Sciences, Poznań, Poland; 6 Department of Bioinformatics and Computational Biology, Poznań University of Medical Sciences, Poznań, Poland; 7 Department of Gastroenterology and Metabolism, Poznań University of Medical Sciences, Poznań, Poland; 8 Department of General and Colorectal Surgery, Poznań University of Medical Sciences, Poznań, Poland

**Keywords:** Sodium butyrate, Diverticulitis, Diverticulosis, Prevention

## Abstract

**Background:**

Microencapsulated sodium butyrate (MSB) has been previously associated with anti-inflammatory and regenerative properties regarding large bowel mucosa. We aimed to examine a role of MSB in patients with diverticulosis, hypothesizing its potential for reduction of diverticulitis episodes and diverticulitis prevention.

**Methods:**

Seventy-three patients with diverticulosis (diagnosed in colonoscopy or/and barium enema or/and CT colography) were recruited for the study and randomized. The investigated group was administered MSB 300 mg daily; the control group was administered placebo. After 12 months, a total of 52 patients completed the study and were subject to analysis (30 subjects and 22 controls). During the study, the number of episodes of diverticulitis (symptomatic diagnosis with acute pain, fever, and leukocytosis), hospitalizations, and surgery performed for diverticulitis were recorded. Additionally, a question regarding subjective improvement of symptoms reflected changes in quality of life during the analysis.

**Results:**

After 12 months, the study group noted a significantly decreased number of diverticulitis episodes in comparison to the control group. The subjective quality of life in the study group was higher than in the control group. There were no side effects of the MSB during the therapy.

**Conclusions:**

MSB reduces the frequency of diverticulitis episodes, is safe, and improves the quality of life. It can play a role in the prevention of diverticulitis.

## Introduction

Approximately 60 % of individuals over age of 60 living in industrialized countries will develop colonic diverticula [1]. Symptomatic disease is likely to occur in 10 to 25 % of that population [2]. Complications of the diverticulitis can be serious and life-threatening including bowel perforation, abscess, fistula, bleeding, and stricture leading to obstruction. Surgical intervention may be warranted and can range from endoscopic or percutaneous procedures to laparoscopic and open surgery. In the USA alone, complications of diverticulitis are estimated for about 130,000 hospitalizations annually [3]. Developing an effective method of prevention of symptomatic diverticulitis in patient with diverticulosis could lead to a significant reduction in both morbidity and mortality.

The aim of our study was to analyze the preventive effect of microencapsulated sodium butyrate (MSB) in patients with diverticulosis in a prospective randomized trial. We used a single closed-end question to analyze for subjective symptom improvement.

## Material and methods

### Study enrollment criteria

Patients qualified for the study reported diverticulosis diagnosed with colonoscopy, barium enema, and/or abdominal CT no earlier than 1 year before the study, with at least one episode of mild or moderate diverticulitis requiring treatment. The exclusion criteria included age <18 years old, previous abdominal surgery (except appendectomy and hernia repair), history of IBD, history of cancer, pharmacotherapy with antibiotics or probiotics within 4 weeks prior to the study, active psychiatric disease, severe comorbidity, pregnancy and breast-feeding, or abdominal symptoms during the last 3 months. In order to avoid bias by dietary changes or tertiary pharmacological agents, the patients were asked not to implement any dietary changes to their typical nutritional habits or use any additional pharmacological treatment throughout the study period.

### Patient population and study design

There were a total of 92 patients recruited to the study. Patients were recruited from two coloproctology outpatient clinics in Poznań, Poland. The study was conducted between June 2011 and November 2012 and was designed as a parallel, double-blinded, randomized, placebo-controlled, per-protocol clinical study, in accordance with the Helsinki Declaration and the regulations of the Ethics Committee of Poznan University of Medical Sciences (26/11) [4]. The study was registered in the EudraCT Trial System with the number 2007-005024-32. During initial visit, patients were informed about the aim of the study and screened for exclusion criteria. Clinical examination was performed and included abdominal examination, body temperature measurement, and WBC count for all patients. At this stage, patients with acute left lower quadrant pain, tenderness, fever (>38 °C), or leukocytosis (WBC > 12,000/mm^3^) were excluded from the study. After this review, 19 patients were excluded from the study, mainly because of the lack of acceptance, planned surgery, or medical therapy in the next 12 months. The remaining total of 73 patients were randomized to a treatment group (*n* = 37) that received MSB (Debutir®, Polfa Łódź) in the dose of 300 mg per day (2 × 150 mg) for 12 months and to a placebo group (*n* = 36) obtaining identical capsule with placebo at identical regimen also for 12 months. Randomization was done based on the numbers of the patient history (odd numbers, control group; even numbers, treatment group). During the study period, patients were advised to report to the outpatient clinic with abdominal pain, fever, or other symptoms associated with inflammation of the diverticula. The diagnosis criteria of the diverticulitis were defined as acute left lower quadrant pain, tenderness, fever (>38 °C), or leukocytosis (WBC > 12,000/mm^3^). In case of mild symptoms of diverticulitis, dietary restrictions with rifaximin and probiotics were implemented. In case of moderate or severe symptoms, abdominal imaging was performed: ultrasound for outpatient visits with assessment diverticulitis grade, as proposed by Mizuki et al. [5], and CT of the abdomen and pelvis following Ambrosetti's criteria [6] in hospitalized patients, in case of substantial ultrasound findings and following inability to obtain conclusive ultrasound images (obesity, intestinal gas). Appropriate surgical intervention was to be performed in most severely affected patients after obtaining a CT scan.

Ultrasound and CT scans were done by the same qualified radiologist, with 15 years of experience in ultrasound (US) and CT of the GI tract. Investigation was done as a blinded study—radiologist had no information about patient randomization (placebo or butyrate group).

**Fig. 1:**
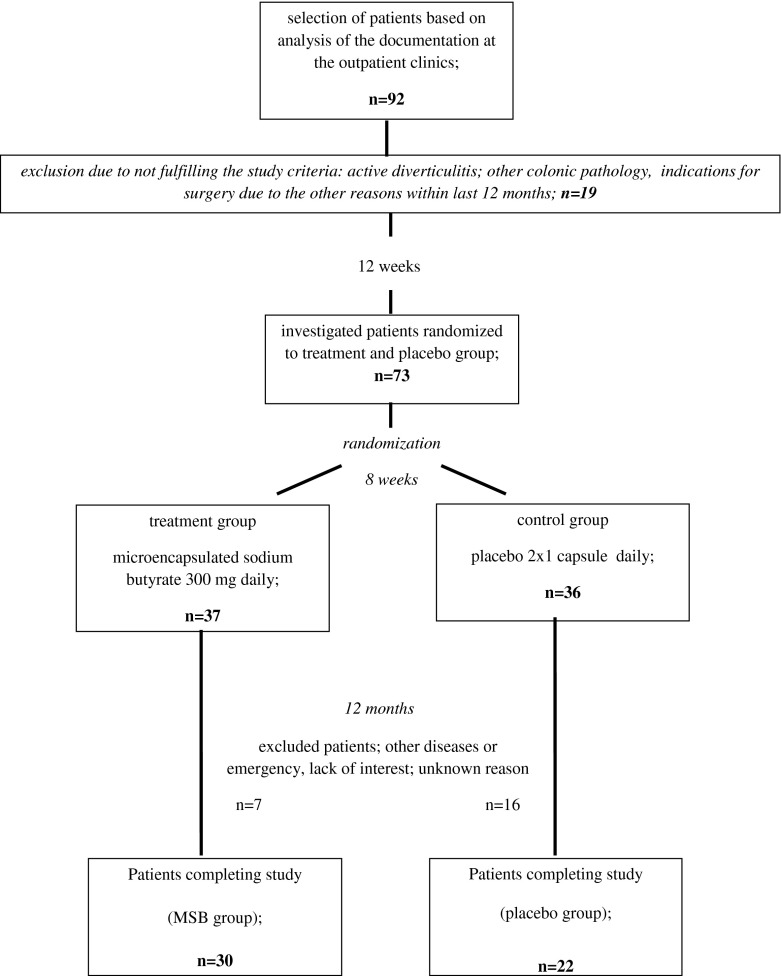
Flowchart representing the course double-blinded randomized study

The study concluded with a final follow-up appointment after 12 months from its initiation. Fifty-two patients reported for the final appointment and completed the study, 30 in the study group and 22 in the placebo group. The main reasons for withdrawal from the study were as follows: lack of interest from the patients, absence during control examinations without reason (14 patients), other diseases without correlation with the gastrointestinal tract (4 patients), moving abroad and lost contact with the patient (2 patients), and car accident and serious injury (1 patient). The course of the study is shown in Fig. 1.

### Evaluation of results

The primary outcome was the number of episodes of diverticulitis experienced by the patients and diverticulitis diagnosed in imaging studies, number of patients hospitalized, and number of patients undergoing surgical intervention for diverticulitis.

The secondary outcome was assessment of subjective symptom improvement with a single closed-end question requiring a “yes” or “no” answer: “Did you observe adequate relief of diverticulosis related to abdominal pain or discomfort within the past 12 months?”

### Statistical analysis

Statistical analysis included medians and quartiles (Q25 and Q75) measuring the degree of dispersion, functioning as median deviation. Additionally, mean values and standard deviations were calculated. The differences between the groups were analyzed with the nonparametric Mann–Whitney test and Kruskal–Wallis test with the Dunn test (post hoc test). Correlation was considered significant at *p* < 0.05.

## Results

Between 2011 and 2012, 92 patients were enrolled in the study. After exclusion of 19 patients who did not meet the criteria of the study, 73 patients were randomized into the treatment group or placebo group. After 12 months, a total of 52 patients concluded the study and their results were analyzed. Baseline demographic characteristics of the investigated patient groups as well as detailed results of our study are presented in Table 1.

**Table 1:** Characteristics of the investigated groups (10 years if diverticulosis and longer) and results of the investigation at 12 months of follow-up

	All patients	Treatment group	Placebo group	*p* value
Number of patients	52	30	22	
Gender (female/male)	30/22	17/13	13/9	
Mean age of patients (years)	64.2	63.7	64.7	ns
SD	6.7	6.09	7.65	
Median	63.5	63	64	
Duration of diverticulosis (years)	9.9	10.47	9.32	ns
SD	4.46	4.18	4.82	
Median	10	8.5	10	
Clinical symptoms of diverticulitis: acute left lower quadrant pain, tenderness, fever (>3 °8C) or leukocytosis (WBC > 12,000/mm^3^), *n* (%)	9 (17.3)	2 (6.7)	7 (31.8)	0.0425
Abdominal ultrasound examinations for suspected diverticulitis, *n* (%)	6 (11.5)	1 (3.3)	5 (22.7)	0.0229
Diagnoses of diverticulitis in abdominal US, *n* (%)	6 (11.5)	1 (3.3)	5 (22.7)	
CT abdomen for suspected diverticulitis, *n* (%)	3 (5.8)	1 (3.3)	2 (9)	ns (low number of events)
Diagnoses of diverticulitis in CT abdomen, *n* (%)	3 (5.8)	1 (3.3)	2 (9)	
Hospitalizations for diverticulitis, *n* (%)	4 (7.7)	1 (3.3)	3 (13.6)	ns (low number of events)
Surgery (Hartmann's procedure) because of diverticulitis, *n* (%)	1 (1.9)	0 (0)	1 (4.5)	ns (low number of events)
Subjective evaluation of the patients (positive answer to the question: *Did you observe adequate relief of diverticulosis related to abdominal pain or discomfort within the past 12 months?*), *n* (%)	22 (42.3)	17 (56.7)	5 (22.7)	0.0143
Number of adverse events, *n* (%)	0 (0)	0 (0)	0 (0)	ns (low number of events)

*ns* not significant

Mean age of patients in the treatment group (*n* = 30) was 63.7 (SD ± 6.09) and 64.7 (±7.65) in the placebo group (*n* = 22). There were more females recruited in the study as well as more females randomized in both arms of the study. Duration of diverticular disease in the treatment group was on average 10.47 years (±4.18) and 9.32 years (±4.82) in the placebo group.

The number of diverticulitis clinical symptom occurrences during 12 months of observation was 2 (6.67 %) in the MSB group and was significantly lower (*p* = 0.0425) than the clinical symptoms of diverticulitis reported in the control group (7 occurrences, 31.8 %). In comparison to the placebo group, the number of abdominal ultrasound examinations performed for suspected diverticulitis and subsequent number of diverticulitis diagnosed based on abdominal US was significantly lower (1 vs 5, *p* = 0.0229). The number of abdominal CT imaging performed for suspected diverticulitis, number of hospitalizations for diverticulitis, and number surgical intervention for diverticulitis were reported to be higher in the treatment group (1 vs 2, 1 vs 3, and 0 vs 1, respectively) but were too low to adequately compare the investigated groups.

Subjective improvement in observed symptoms based on a single closed-end question (yes or no answer): “Did you observe adequate relief of diverticulosis related to abdominal pain or discomfort within the past 12 months?” was reported to be higher in the treatment group (55.67 vs 22.73 %, *p* = 0.0143).

At the time of the study as well as after the conclusion of the study, there were no adverse or side effects of MSB or placebo observed.

## Discussion

In the present study, we sought to determine the protective effect of MSB on incidence of symptomatic diverticulitis in patients with diverticulosis. To determine MSB efficacy, we designed a randomized placebo-controlled study recording rates of clinical symptoms related to diverticulitis as well as patients' subjective impression of symptom improvement over the 12 months. As far as we are aware, our study is the first to assess the value of MSB in the reduction of symptomatic diverticulitis occurrence.

Diverticulitis is not only responsible for increasingly common cause of hospital admissions [7] but also poses a risk of continued episodes of diverticulitis in patients' lifetime. It has been estimated that within 5 years after a successful nonsurgical treatment, up to 80 % diverticulitis can reoccur, leading to impaired quality of life and physical activity with increased costs due to multiple specialist consultations, pain medication, and work absence [8–11].

In order to reduce incidence and recurrence, various methods of diverticulitis prevention have been proposed. Appropriate diet changes including high-vegetable and high-fiber intake and has been proven effective in previous studies [12, 13]. The protective effect of dietary fiber has been explained by increased bulk of stool, distention of colonic lumen, and a decline in intraluminal pressures, thus reducing colonic transit time [14, 15]. In patients with already developed diverticular disease and patients who are symptomatic, evidence of benefit of increased fiber intake seems less established. Apart from dietary changes, reduced body mass as well as an inverse association between vigorous physical activity and occurrence of diverticular disease has been demonstrated [16].

With regard to pharmacological approach, an emergent role of cyclic nonabsorbable antibiotic has been extensively investigated. Increased efficacy of rifaximin in diverticular disease symptom reduction when compared to simple dietary fiber supplementation has been established [17]. A study by Tursi et al. has demonstrated a significant advantage of combined efficacy of rifaximin plus mesalazine against rifaximin alone in observed severity of symptoms, improved bowel habits, and diverticulitis recurrence [18].

Administration of probiotics is known to reduce incidence of inflammatory disease of the bowel and has been associated with benefit in symptomatic diverticulosis [19–21]. The probiotics act by influencing antimicrobial agent production and interact competitively for metabolites with pro-inflammatory organisms. The combination of *Lactobacilli* spp. with rifaximin seems effective in the reduction of severe diverticulitis and can prevent recurrences, thus minimizing the need for surgical treatment [20].

In the current study regarding diverticulitis occurrence reduction, we utilized MSB, a short-chain fatty acid previously proven to provide symptomatic relief in patients suffering from various range of colonic diseases, such as IBS, inflammatory bowel disease, diarrhea, and malabsorption and suggestive of preventive role in cancerogenesis of colonocytes [22–24]. While there is no certainty as to what renders sodium butyrate (SB) to be so beneficial in such a wide range of colonic diseases, it has been established that SB can act as a regulator of intestinal environment. It is a preferred energy substrate for colonocytes, can moderate intestinal permeability, reduce oxidative stress, and reinforce colonic defense barrier leading to decreased inflammation of mucosa, increased cell regeneration rate, and promote healing [25–27]. What prompted the authors to study the efficacy of SB for diverticulitis was the lack of side effects of SB [28, 29] and similarities between diverticulitis and irritable bowel syndrome for which SB has been shown effective, such as presence of abnormal colonic motility, visceral hypersensitivity, presence of low-grade inflammation, and increased circulating levels of either substance P or vasoactive intestinal polypeptide [30, 31].

We utilized a microencapsulated form of SB to maximize the biological effect on the colon. Unprotected forms of SB induce fast absorption in the small bowel, thus preventing its passage to the large intestine where local release of SB seems to be of most benefit. In our study, we utilized a unique lipid membrane microcapsulation, designed to release SB distal from the ileo-cecal region and used successfully in previous studies [22]. Other formulas to release SB in the colon have been used, including hydroxylpropyl methylcellulose and Shellac coating [32].

Several disease-specific questionnaires based on the clinical symptoms are used to evaluate GI symptoms in patients with diverticulitis [33]. These can be time-consuming, which can be a limitation in an efficiency-oriented clinical practice. There are questionnaires based on a single closed-end question providing an efficient measurement of subjective colonic symptom improvement, previously validated for other diseases such as IBS. The validated IBS questionnaire utilizes a question: “Did you observe adequate relief of irritable bowel syndrome symptoms related to abdominal pain or discomfort within the past week?” [34]. Although the result is not qualitative, it seems to adequately reflect the efficacy of treatment as seen by patients. Prior to our study, we were seeking for a simplified assessment of symptom improvement for diverticulitis. Based on IBS symptom improvement question, and taken similar symptoms related to disease, we established a modified question for symptom improvement related to diverticulitis. The relief of symptoms was investigated using a single closed-end question: “Did you observe adequate relief of diverticulosis related to abdominal pain or discomfort within the past 12 months?” In comparison to the placebo group, we recorded improved relief in the studied group within the treatment group. In our opinion, it is a very important assessment tool of the implemented therapy that reflects its combined efficacy. This type of validation can be useful in daily clinical practice to simplify the validation procedure. This instrument is a validated and generally accepted simple outcome assessment of IBS treatment efficacy [34, 35]. We are, of course, aware of the large simplification methods for assessing performance through lack of validated life quality questionnaire.

Overall, our findings demonstrate that diverticulosis patients administered MSB regimen suffer from a smaller number of episodes typical of diverticulitis than the placebo group (6.67 % vs 31.8 %, *p* = 0.0425). We postulate that this reflects the preventive properties of MSB in this group of patients. With regard to a single closed-end question for symptom assessment, we recorded subjective improvement in comparison to the placebo group (56.67 vs 22.73 %, *p* = 0.0143). This can further implicate increased quality of life in patients with diverticulosis upon MSB regimen. From practical standpoint, this could translate into future implementation of MSB in patients with diverticulosis. In smaller scale, preventive therapy could be applied alone or with other agents for groups at higher risk of recurrence, which have previously been identified and include patients with family history of disease, long segment of diseased colon, and history of retroperitoneal abscess at first presentation [24].

Current analysis has several limitations. Increased power of study would be beneficial, especially in the context of events that were not frequent and did not reach enough power to attain statistical significance. Diagnosis criteria of diverticulitis are based on clinical symptoms which are subjective to the physician's individual assessment and experience, thus making standardization and comparison difficult. Although the question for subjective clinical symptom improvement seemed practical, it has not been validated for diverticular disease. Lastly, the authors feel that a larger study with multi-institutional input would be valuable.

## Conclusions

Administration of MSB in asymptomatic patients with diverticulosis can lead to significant decrease of clinical diverticulitis incidence (*p* = 0.425). In addition, it can decrease the need for imaging studies associated with diverticulitis (*p* = 0.0229). After a 12-month course of MSB in patients with diagnosed diverticulosis, a subjective improvement in occurrence of clinical symptoms associated with diverticular disease was reported (*p* = 0.0143). No adverse effects associated with MSB administration have been observed. Management of diverticulosis with MSB is safe and can play an important role in the prevention of progression to clinical diverticulitis.
